# Aging-Induced Biological Changes and Cardiovascular Diseases

**DOI:** 10.1155/2018/7156435

**Published:** 2018-06-10

**Authors:** James Oluwagbamigbe Fajemiroye, Luiz Carlos da Cunha, Roberto Saavedra-Rodríguez, Karla Lima Rodrigues, Lara Marques Naves, Aline Andrade Mourão, Elaine Fernanda da Silva, Nabofa Enivwenaye Egide Williams, José Luis Rodrigues Martins, Romes Bittencourt Sousa, Ana Cristina Silva Rebelo, Angela Adamsk da Silva Reis, Rodrigo da Silva Santos, Marcos Luiz Ferreira-Neto, Gustavo Rodrigues Pedrino

**Affiliations:** ^1^Biological Sciences Institute, Federal University of Goiás, Goiânia, GO, Brazil; ^2^Center for Studies and Toxicological-Pharmacological Research, Faculty of Pharmacy, Federal University of Goiás, Goiânia, Brazil; ^3^University Center of Anápolis-Unievangélica, 75083-515 Anápolis, GO, Brazil; ^4^Department of Basic Sciences, Universidad Santo Tomas, Campus Osorno, Osorno, Chile; ^5^Department of Physiology, Ben-Carson (Snr) School of Medicine, Babcock University, Nigeria; ^6^Department of Natural Sciences, Special Academic Unit of Human Sciences, Federal University of Goiás, Goiânia, Goiás, Brazil; ^7^Laboratory of Experimental Physiology, Faculty of Physical Education, Federal University of Uberlândia, Uberlândia, Minas Gerais, Brazil

## Abstract

Aging is characterized by functional decline in homeostatic regulation and vital cellular events. This process can be linked with the development of cardiovascular diseases (CVDs). In this review, we discussed aging-induced biological alterations that are associated with CVDs through the following aspects: (i) structural, biochemical, and functional modifications; (ii) autonomic nervous system (ANS) dysregulation; (iii) epigenetic alterations; and (iv) atherosclerosis and stroke development. Aging-mediated structural and biochemical modifications coupled with gradual loss of ANS regulation, vascular stiffening, and deposition of collagen and calcium often disrupt cardiovascular system homeostasis. The structural and biochemical adjustments have been consistently implicated in the progressive increase in mechanical burden and functional breakdown of the heart and vessels. In addition, cardiomyocyte loss in this process often reduces adaptive capacity and cardiovascular function. The accumulation of epigenetic changes also plays important roles in the development of CVDs. In summary, the understanding of the aging-mediated changes remains promising towards effective diagnosis, discovery of new drug targets, and development of new therapies for the treatment of CVDs.

## 1. Introduction

**Figure 1:**
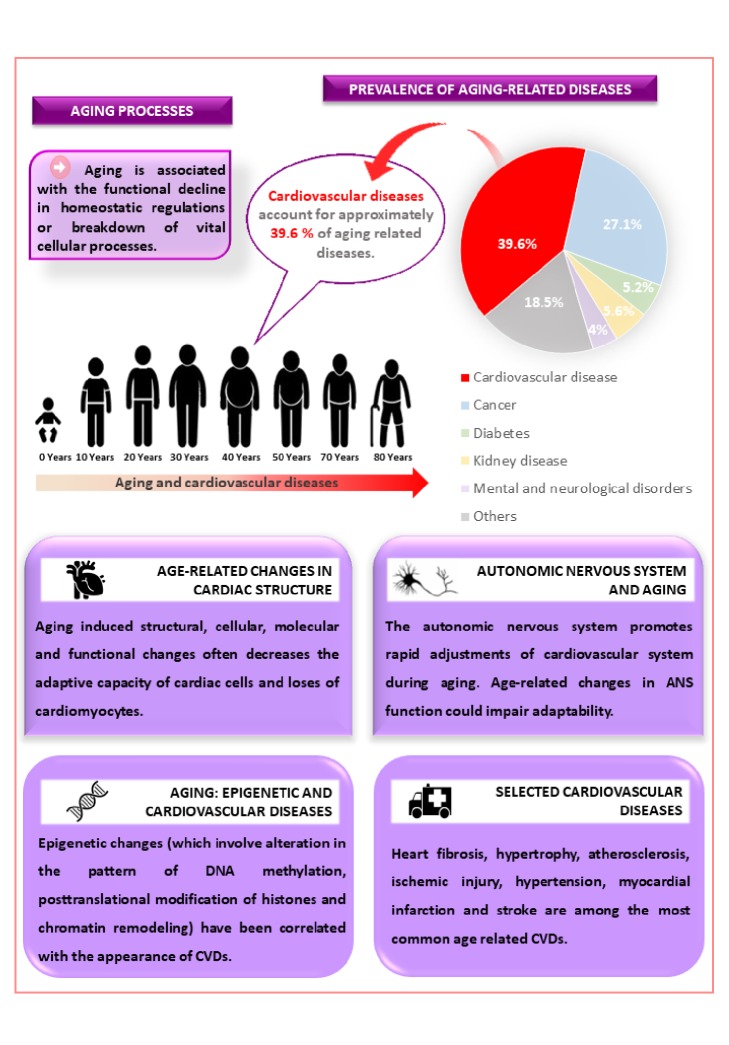
Aging processes, prevalence of aging-related diseases, and some selected cardiovascular diseases.

Cardiovascular diseases (CVDs) which are responsible for over 4 million annual deaths in Europe remain one of the leading causes of death worldwide [1]. Currently, there is an increase in annual cases of CVDs such as heart fibrosis, hypertrophy, atherosclerosis, ischemic injury, hypertension, myocardial infarction, and stroke which, put together, account for approximately 39.6% of age-related diseases (Figure 1). Aging reduces the efficiency of homeostatic regulation thereby promoting an increase in tissue damage, rate of morbidity, and mortality [2–4].

Aging involves changes in the complex regulatory interplay among cells, organs, and systems [5]. Cardiac and smooth muscle cells participate in involuntary control of heart and vascular functions. The integrity, excitability, conductivity, contractility, and elasticity of these cells are important to cardiovascular control. Progressive loss of physiological function of cardiomyocytes and vascular smooth muscle cells have been associated with cellular aging [6].

Cells undergoing aging processes have a way of communicating their internal status to adjacent cells [7–9]. At the cellular level, aging could disrupt trophic and metabolic signaling pathways prior to the loss of cardiac and vascular functions. In addition, autonomic nervous system (ANS) dysregulation and complex epigenetic changes also induced CVDs [10, 11].

Recently, Steenman and Lande (2017) [12] correlated aging population and increasing prevalence of CVDs. According to these authors, human cardiac aging establishes common pathways with heart disease. Cardiac aging is often characterized by functional, structural, cellular, and molecular changes. The understanding of cardiac aging may unravel heart pathophysiology and promotes effective treatment of CVDs. The detailed reviews on the neurohormonal signaling and morphofunctional changes in diastolic, systolic, and electrical functions induced by aging have been extensively reported [12]. In the present review, we focused on the cardiac aging and autonomic and epigenetic changes underlining some selected CVDs.

## 2. Age-Related Changes in Cardiac Structure

Aging-related structural modifications in the cardiovascular system often interfere with the functional and adaptive capacity of the heart and vessels [13]. In humans, cardiac aging is associated with left ventricle hypertrophy, fibrosis, and diastolic dysfunction, resulting in reduction of diastolic filling and cardiac output ejection fraction [14, 15]. Although the underlining mechanisms are yet to be fully unravelled, studies suggest that cardiomyocyte apoptosis and vascular stiffness were associated with aging-induced structural and functional modifications [16, 17].

The cardiac hypertrophy in response to aging and other physiological stimuli maintained functional demands of the heart in a compensatory manner. However, excessive heart demands could make hypertrophy a pathological condition [18]. Older hearts were characterized by the thickening of left ventricular wall due to an increase in cardiomyocyte size [19], asymmetric growth of the interventricular septum, and change in the heart shape [20]. These alterations could reduce or increase contractile efficiency of the heart.

Clinical and preclinical reports have associated biochemical changes with cardiac hypertrophy. The extracellular signal-regulated kinase 1/2 (ERK1/2) played a pivotal role in the development of cardiac hypertrophy through Ras/Raf/MEK/ERK signaling pathways [21, 22]. Moreover, the protein kinase B, Akt, or mTOR among other downstream regulators have also been implicated in cardiac hypertrophy [22, 23]. Manne et al. (2014) [22] earlier reported an association between the activation of ERK1/2 and Akt signaling pathways to cardiomyocyte hypertrophy. It thus appears that these signaling pathways regulate the aging-related cardiac hypertrophy.

At the microscopic level, cardiac hypertrophy is associated with a high loss of myocytes. Earlier studies indicate that the number of ventricular myocytes was reduced with aging as a result of apoptosis [24–28]. Olivetti et al. (1995) [24] reported a loss of about 45 million myocytes per year in the left ventricle of aging men. Although aging is accompanied by cardiomyocytes loss, studies have shown an increase in the ventricular myocyte volume [19, 24]. Thus, it was hypothesized that the age-related myocyte loss can increase the mechanical load on the remaining myocytes, resulting in compensatory hypertrophy. In addition to the reduction in the cardiomyocytes number, the peripheral vascular stiffening may contribute to progressive hypertrophy in aged hearts. The loss of aortic elasticity increased the mechanical load on the heart and accelerated heart failure. The hemodynamic overload, caused by aging-related arterial stiffening, contributed to the left ventricle myocyte hypertrophy [17] and increase of collagen deposition in the cardiac tissue. Therefore, hypertrophy appears to be an adaptive mechanism to maintain cardiac function in response to aging-induced structural changes in the cardiovascular system.

Other aging-induced structural modification included epicardial adipose tissue deposition [29, 30] and calcification of the aortic valve leaflets that were associated with atherosclerosis and heart failure [31]. Additionally, the aging heart was characterized by the proliferation of cardiac fibroblasts [32]. This proliferation may result in the accumulation of collagen prior to atrial and ventricular fibrosis in the elderly [33, 34]. Over the years, aging-induced myocardial fibrosis has been studied in human [35, 36], rodent [37, 38], dog [39], and sheep [40].

Aging increased elements of the cardiac extracellular matrix such as glycoproteins, proteoglycans, glycosaminoglycans, integrins, and collagen [41]. It has been reported that collagen content in the left ventricle area of mice also increased from 1-2% to 2-4% [38]. Autopsies of elderly subjects showed an increase in collagen type I and a decrease in collagen type III when compared with younger subjects [42]. Collagen type I had higher tensile strength, whereas collagen type III was more distensible. For that reason, a higher ratio of collagen type I may contribute to left ventricle stiffness and impair cardiac biomechanical functions [35, 41, 42]. In addition, these changes in the extracellular matrix around myocytes or myofibrillar bundles inhibited the propagation of electrical signals, resulting in arrhythmias [7, 43].

The increases in collagen with aging involved posttranscriptional events [41]. Synthesis and breakdown of the extracellular matrix determined high collagen levels. In aged hearts, the stimulation of cardiac fibroblasts by fibrogenic growth factors (such as TGF-*β*) induced synthesis of matrix proteins and protease inhibitors, leading to fibrotic remodeling and, consequently, diastolic and systolic dysfunction [44, 45]. In addition, reduction in the evolutionarily conserved intracellular autophagy pathway in the heart with aging often triggers structural and functional cardiovascular dysfunctions [46].

Autophagy is an important intracellular process that controls lysosomal degradation of pathogens, aged or damaged proteins, and organelles to protect cells [47]. In the heart, autophagy played an essential role against structural and functional dysfunction during basal state and hemodynamic stress [48]. However, the autophagy machinery becomes susceptible during the aging, resulting in an inadequate performance of cardiac activity [23]. Therefore, the reduction of autophagy with advanced age can affect the capacity to recycle and degrade damaged intracellular components, thereby leading to structural and functional alterations [49]. The serine/threonine protein kinase negatively regulates autophagy through mTOR complex 1 [50]. Aging reduced cardiac autophagy has been associated with overactivation of Akt [23].

The autophagy inhibition shortens lifespan and exacerbates aging-associated cardiomyopathies [46]. The rapamycin-induced autophagy (rapamycin as an inhibitor of mTOR signaling) extended longevity [51] and promoted cardiac performance such as improvement of ejection fraction and reduction in ventricular hypertrophy in aged mice [52].

## 3. Autonomic Nervous System (ANS) and Aging

The control and integration of the cardiovascular system is characterized by a complex interaction among the heart, kidney, brain, vasculature, and endocrine systems through intrinsic and extrinsic mechanisms. The extrinsic mechanisms are dependent on the ANS and the endocrine system that facilitate rapid adjustments of the cardiovascular function [53–55]. Age-related changes in ANS function could impair adaptability of an elderly individual to the environment [56]. Normally, aging increases plasma catecholamine concentrations and sympathetic nerve activity [57, 58]. Recent studies have suggested that aging-induced increase in sympathetic tone to skeletal muscle vasculature is devoid of decline in respiratory-sympathetic coupling [59]. Hence, the elevation of muscle sympathetic nerve activity in an aged individual could result in endothelial malfunction and arterial stiffness [60]. Aging has been implicated in the impairment of *α*-adrenergic receptor sensitivity and vascular responsiveness [61]. A higher decrease in mean arterial pressure of older women in response to autonomic blockade as compared to younger women demonstrates the importance of the ANS in maintaining blood pressure in elderly individuals [62]. Reports have shown that age-related alterations in autonomic nerve activity reduced blood pressure, cerebral blood flow, bladder function, and heart rate variability (HRV) [54, 63, 64].

HRV is an indicator of arrhythmic complications and strong predictor of mortality and sudden death [65]. The analysis of HRV provides vital information on the contributions of the ANS to the consecutive oscillations of heart rate [63, 66]. Nocturnal reduction in cardiac parasympathetic activity in elderly individuals elicits a decline in cardiovagal control [65]. Aging could disrupt ANS through reduction and increase in the input of parasympathetic and sympathetic nervous systems, respectively [67–69]. The parasympathetic and sympathetic imbalance decreases HRV [70, 71] which in turn promotes the incidence of cardiovascular events [67]. The high frequency index of parasympathetic modulation indicated a relationship between aging and decline in HRV. The low frequency index of HRV has been associated with an increase in sympathetic modulation. The increase in heart rate combined with the HRV reduction contributes to the degeneration of cardiac autonomic function during aging [72].

Reports have linked CVDs to morbidity and mortality among postmenopausal and obese women (40-55 years) around the world [73–77]. Some studies have reported higher values of HRV in premenopausal women as compared to postmenopausal women of the same age group. The cardioprotective contributions of sex hormones in such women have been reported [78, 79]. According to Davy et al. (1998) [80], young women had higher HRV in comparison with menopausal women. Studies have shown higher prevalence age-dependent HRV reduction among men [72].

Earlier reports on epigenetic relevance in aging-induced CVDs suggested that an active lifestyle is important to the health of the elderly. Elderly people were considered to be prone to slower cardiac, metabolic, and autonomic response as compared to younger ones due to deceleration in vagal reactivation and impairment of cardiac autonomic modulation [81]. The deleterious effects of advanced age on autonomic regulation could be minimized by intensive exercise [82, 83]. Intensive exercise could increase muscle mass and strength without changes in cardiovascular function [82]. A physical training regime could improve physiological adaptations and autonomic function [84]. Although intensive exercise seems to be beneficial to older individuals, there are needs for further research that could lead to health-enhancing exercise programs designed.

The ANS abnormalities were thought to be a common underlying pathophysiology of CVDs such as hypertension and heart failure [85]. In this regard, the atrial fibrillation (AF) is the most frequent arrhythmia that was associated with the imbalance of sympathetic and parasympathetic drive to the heart. Patients with AF had reduction in cardiac performance due to the loss of the atrial contraction and ventricular disorder. The incidence of AF increases dramatically during aging [86, 87]. The surgical ablation which involves various degrees of denervation of ANS has been shown to be efficient against AF [88, 89]. The experimental procedures that target autonomic imbalance in animal models and human studies of AF have been developed [90]. Studies using a high frequency for sympathetic-mimicking atrial stimulation [91] and radiofrequency ablation of the cardiac autonomic ganglion plexus were acclaimed to be a good procedure for the abolishment of AF [92]. Nevertheless, in human patients, studies of combined ganglion plexus destruction and pulmonary vein isolation by radiofrequency ablation promoted reasonable results in preventing AF [93, 94]. On the other hand, pulmonary vein isolation plus renal denervation improved the lifespan by 1 year when compared to pulmonary vein isolation alone [95].

## 4. Relevance of Epigenetics and Advanced Age in the Evolution of Cardiovascular Diseases

Advanced biological age is often characterized by the accumulation of epigenetic changes that can be correlated with the appearance of CVDs [96]. Chronic stress is one of the major environmental factors responsible for epigenetic changes that affect the cardiovascular system. Evidence has shown that chronic stress promotes modification in the hypothalamic pituitary adrenal pathway [97, 98].

Previous work of Natt et al. (2015) [98] showed repetitive stress-induced decrease in DNA methylation. Kim and Stansfield (2017) [99] inferred that changes in the patterns of acetylation and methylation of genes encoding MMPs were associated with the development of aorta aneurysms. Galán et al. (2016) [100] reported an increase in the expression of histone deacetylase (HDAC) during aortic aneurysm and that administration of class 1 and 2 HDAC inhibitors resulted in a reduction of aortic aneurysm in mice. These results support epigenetic mechanisms of aortic aneurysm.

In addition to aortic aneurysm, cardiomyopathy, a pathology marked by intrinsic myocardial weakness, contractile dysfunction, and congestive heart failure, is one of the main CVDs induced by poor nutrition and a sedentary lifestyle. The development of cardiomyopathy has been associated with the appearance of mitochondrial cardiac polymerase dysfunction and an increase in the methylation of the cardiac DNA. Koczor et al. (2016) [96] correlated cardiomyopathy to the mitochondrial DNA depletion. The authors reported different methylation patterns between the hearts of old and young rats.

The synthesis and excessive accumulation of extracellular matrix proteins towards healing of lesions in the heart muscle have been identified as one of the causes of heart failure. Ghosh et al. (2016) [101] identified microRNAs as important biomarkers for the development of this pathophysiological state. MicroRNAs participate in the regulation of various genetic and epigenetic mechanisms. Structural or epigenetic modifications in these microRNAs have been linked with aging [102].

## 5. Atherosclerosis

Cardiovascular system disorder such as atherosclerosis is common among aged patients [103]. Atherosclerosis is a multifactorial and progressive disease. Its etiology involves the accumulation of lipid, inflammatory cells, fibrosis elements, and plaque formation and deposition in the arterial walls [104–106]. The aging process could accelerate structural and compositional modifications observed in atherosclerosis. Spatial increase in the vessels and intimal and medial layers thickening are among such modifications. In addition, the accumulation of an extracellular matrix rich in glycosaminoglycans, collagen, and elastin fibers in the vasculature has been attributed to aging [107].

As reported in literature, endothelial cell injury and atherosclerosis clearly suggest the susceptibility of aged vessels to lesion [108–110]. Previous experiments that compared old and young rabbits subjected to long period of hyperlipidemic diet showed that old rabbit arteries constantly develop fibroatheromatous plaques [109]. The formation of plaques, cholesterol deposits (atheroma) with a fibrous cap (sclerosis), characterized the inflammatory process of atherosclerosis. The infiltration of subendothelial spaces of arteries by oxidized lipoprotein often initiated atherosclerosis [111]. Kolodgie et al. (2007) [112] correlated atheroma to pathological thickening of the intima, loss of vascular smooth muscle cells, lipid deposition, and macrophage infiltration. The vascular remodeling reinforces the characterization of aging vessels by thickening and loss of elasticity [113]. The cellular changes in atherosclerosis disease could reduce the number of medial vascular smooth muscle cells and increase collagen deposition [114, 115].

Some authors have addressed the implications of cellular senescence in the atherosclerosis process [116–118]. The cellular senescence could occur in two forms: (i) replicative and (ii) stress-induced premature senescence. The replicative one arises from DNA damage-induced telomere shortening. This damage could result from the high content of reactive oxygen species (ROS), oncogenes, and telomere [117]. Biomarker such as senescence-associated *β* galactosidase (SA*β*G) was found during senescence of human cells [119]. A high amount of SA*β*G-positive in atherosclerotic lesions and old vessels reaffirmed the link between atherosclerosis and senescence [120]. In addition, the pathogenesis of atherosclerosis also involves the recruitment of immune cells. At the site of a lesion with abnormal functioning of endothelium, leukocytes, vascular smooth muscles, and platelets constitute the atheroma. As lipid peroxidation occurs, many molecules that control cell proliferation are released. Moreover, the endothelial cells recruit monocytes and macrophages through the release of colony-stimulating factors [121]. The monocytes and macrophages scavenge potentially harmful compounds. However, the inflammatory factor released by these cells promotes extracellular matrix protein deposition and changes of vascular smooth muscle cells proliferation and migration [122, 123].

Some experimental models of atherosclerosis have associated nutrition to age-induced vascular changes [124]. In these experiments, a modifiable diet that has beneficial effects on old vessels was used to target caloric restriction (CR) [125]. CR has been reported as a dietary intervention for promoting longevity and delaying age-related diseases, including atherosclerosis [126]. Previous study had implicated nicotinamide adenine dinucleotide- (NAD-) dependent deacetylases and adenosine diphosphate-ribosyltransferases (e.g., SIRT1) in the beneficial effect of CR [117]. According to Kitada et al. (2016) [126], CR-induced SIRT1 has an antiaging property. Hence, this molecule could be an important pharmacological target against atherosclerosis.

A possible mechanism by which CR exerts such beneficial effect could involve the actions of sirtuins, particularly SIRT1. SIRT1 has been regarded as a longevity gene that protects cells against oxidative and genotoxic stress [127]. The activation of SIRT1 could exert many physiological effects, including reduced apoptosis, enhanced mitochondrial biogenesis, the inhibition of inflammation, the regulation of glucose and lipid metabolism, and adaptations to cellular stresses such as hypoxia, endoplasmic reticulum (ER) stress, and oxidative stress [126]. Thus, SIRT1 may exert protective effects against vascular aging and atherosclerosis.

Recent studies have shown that SIRT1 could be a regulatory target of multiple miRNAs, such as miR-33, miR-34a, miR-221/222, miR-217, miR-132, and let-7g. Among these miRs, miR-34a has been implicated in the expression of SIRT1 in vascular endothelial cells. Aging endothelial cells expressed high levels of miR-34a and low levels of SIRT1. In addition, earlier reports also indicated an overexpression of miR-34a and increased acetylated p53 levels in endothelial cells. Taken together, these results suggest that miR-34a regulates endothelial senescence in part through decreases of SIRT1 [128, 129].

Earlier report showed that miR-33a/b family played an important role in posttranscriptional repression of the ATP-binding cassette transporter A1 (ABCA1). This role is essential for the biogenesis of high-density lipoprotein (HDL) and reversal of cholesterol transport from peripheral tissues to the liver. The genetic deletion or anti-miR-mediated inhibition of miR-33 in mice has led to the depression of hepatic ABCA1 and up to 40% increase in circulating HDL [130]. This result suggested that miR-33 silencing could be a useful therapeutic strategy against atherosclerosis [131]. Ouimet et. al. (2017) [132] described miR-33 regulation of autophagy in atherosclerosis. The authors reported that the treatment of atherosclerotic Ldlr −/− mice with anti-miR-33 restored defective autophagy in macrophage foam cells and plaques and promoted apoptotic cell clearance to reduce plaque necrosis.

Recent advances in cellular research have suggested new possibilities for the pharmacological treatment of atherosclerosis [118, 133]. The use of antigens as vaccines [134], immunosuppressors (cyclosporine and sirolimus), and anti-inflammatory drugs has been proposed for the treatment of atherosclerosis. However, anti-inflammatory drugs such as Rofecoxib (cyclooxygenase-2 inhibitor), which may trigger cardiovascular problems [135], require a cautious application in patients with CVDs [136]. Hence, better therapies are still needed for atherosclerosis treatment. New anti-inflammatory agents are under development and it is hoped that they will be available in the future [137].

## 6. Stroke

Stroke is the second most frequent cause of death after ischemic heart in the developed countries [138] and commonly occurs in individuals over 65 years [139, 140]. Stroke may be caused by brain cell damage and brain tissue ischemia due to the disruption of blood supply by a thrombus [141]. It has been reported that the incidence of stroke increases with age [142].

At midlife, neuronal atrophic and glial cell changes begin at the same time. These changes result in the degeneration of white matter. Aging-induced modifications of white matter can influence the susceptibility of axons to ischemia [113]. The astrocytic, microglial hyperactivity and subsequent development of leukoaraiosis have been reported. According to Koton et al. (2009) [143], leukoaraiosis could occur in up to 44 % of stroke patients. As observed in the brains of old rats, a reduction of Na^+^–K^+^-ATPase performance leads to damage of the integrity of brain cell membranes and causes white matter vulnerability to ischemia [144]. The increase in glutamate concentration can trigger an influx of calcium ions, excitotoxicity, and cell death [145].

The brain microvasculature also suffers from the influence of the aging process. The structural and functional degeneration of the blood-brain barrier (BBB) during aging could disrupt local perfusion [146, 147]. The changes in pH, water content, and the accumulation of glutamate and lactate in brain interstitial fluid are associated with the reduction in cerebral blood flow (CBF) [148–150]. Cerebral hypoperfusion can induce microcirculation disturbance and oligemia as well as cerebral endothelium loss devoid of ischemic injury [149, 151]. Some experimental data have suggested that the modifications of white matter brain microvasculature could lead to leukoaraiosis [152–154].

Despite the fact that BBB has the ability to adjust to slight aging-related alterations, its permeability often increases [155, 156]. Cerebral vessel alterations during aging may decrease cerebrovascular reservoirs and make the brain more prone to ischemic injury and vascular insufficiency [151]. All these changes could result in an ischemic stroke and vascular cognitive deficiency in the elderly [142]. Age-related alterations that lead to a dysfunctional phenotype [157] suggest the primary effect of aging in the development of CVDs [158]. The dysfunctional endothelial phenotype could induce hemodynamic changes in humans, nonhuman primates, and rodents [157, 159, 160]. The arterial endothelium participates in vital autocrine and paracrine functions that regulate fluid state, blood-tissue exchange of molecules, vascularization, immune system response, and vascular resistance. The resistance arteries [161] maintain a healthy vascular endothelium state through chemical mediators like pro- and antioxidants, vasodilators, vasoconstrictors, and pro- and anti-inflammatory molecules, among others.

Aging can be associated with endothelial dysfunction or impaired endothelial-dependent dilation (EDD). Earlier, Widlansky et al. (2003) [162] demonstrated that increased age was the strongest independent correlate of EDD [163]. Thus, it could be assumed that vascular aging is critical to the development of age-related CVDs. Findings from studies involving vascular aging models have also demonstrated impaired EDD, permeability, angiogenesis, and fibrinolysis [164–166]. Risk factors, such as elevated blood pressure, LDL cholesterol, blood glucose, and sodium intake [167–171], can modulate the severity of endothelial dysfunction. The clearance of high postprandial glucose and lipids decreases with age [172, 173]. In addition, studies that involved endothelial culture which reported physiological elevations in glucose and lipids corroborate the vascular aging phenotype [174, 175].

## 7. General Discussion and Perspective

Although aging and CVDs fall into a broad field of research with several publications, it is very difficult to cover all the relevant literature on their probable mechanistic connections. The established link between aging and biological changes has greatly advanced our knowledge about diseases such as heart fibrosis, hypertrophy, atherosclerosis, ischemic injury, hypertension, myocardial infarction, and stroke. The current review was narrowed down to the alterations at the organ (heart or cardiac tissues), cellular, molecular, and ANS levels in an attempt to connect the complexity of aging processes to the development of CVDs. Interestingly, some aging-related CVDs have been discussed and published in recent times [176–178].

According to Wang et al. (2010) [179], cardiac remodeling which is an adaptive process often leads to electrical instability, ventricular arrhythmia, cardiac dysfunction, and the death of cardiomyocytes. The incidence of cardiac dysfunction, arrhythmogenesis, and myocardial infarction has been linked to an increase of sympathetic excitation during the remodeling of ANS function as seen in the starred ganglia and dorsal root ganglia [180]. The ganglionic compromise of the autonomic plexuses during adolescence increases susceptibility to atrial fibrillation, micro- and macrostructural changes in the atrial myocardium, intracellular damage (myocyte degeneration, apoptosis), and extracellular fibrotic proliferation [181].

The ANS is predominantly an efferent system that transmits impulses from the central nervous system (CNS) to the periphery. Its physiological roles among others include control of heart rate (HR), force of heart contraction, constriction, and dilation of blood vessels. The functions of autonomic nerves are mediated through the release of neurotransmitters that bind to specific cardiac and vascular receptors. The evolution of CVDs has been associated with alterations in ANS control mechanisms. Advanced age is often characterized by structural, biochemical, and functional changes in the arterial system. In the previous report, some of these changes were associated with epigenetic modifications through DNA methylation, histone modifications, and anomalous gene regulation [99]. These changes could gradually lead to abnormal cardiac and vascular structures prior to the development of CVDs.

The ANS controls cardiac functions by activating its efferent sympathetic nerves to increase heart rate and cardiac contractility [182]. In addition, the parasympathetic nerves exert control over heart functions through direct vagal mediated bradycardia [183, 184]. Recordings from sympathetic and parasympathetic nerves suggest that aging might enhance basal norepinephrine and decrease acetylcholine levels, resulting in depression of heart rate variability [63, 64, 185]. Taken together, these evidences indicated that the imbalance of sympathetic and parasympathetic drive to the heart could promote a direct cardiac dysfunction during aging. On the other hand, cardiac marker such as left ventricular hypertrophy (LVH), an adaptive process that occurs in response to peripheral hemodynamic load, was reported as a major independent risk factor for cardiovascular morbidity and mortality in aging population [186]. In fact, the LVH in hypertensive patients correlated to higher risk of developing systolic heart failure [187]. Thus, it is difficult to determine whether age-induced cardiac dysfunction is closely associated with direct effects or through peripheral hemodynamic load on the heart. Therefore, the combination of these two factors is likely to result in morphofunctional changes in aging heart.

According to Zhang et al., 2014 [188], the evaluation of human longevity correlated lifespan and ALDH2 gene mutation. Recent work showed that ALDH2 regulated autophagic activity and played important roles in cardiac aging. For instance, the overexpression of ALDH2 suppressed autophagy and induced myocardial dysfunction in the previous study [189]. According to Wu et al. (2016) [190], the ablation of ALDH2 may lead to cardiac aging. The authors argued that aged hearts showed a significant decrease in ALDH2 activity. It was suggested that a decrease in the activity of this enzyme caused an accumulation of 4-HNE and carbonyl proteins which in turn compromises autophagic processes and cardiac function.

**Figure 2:**
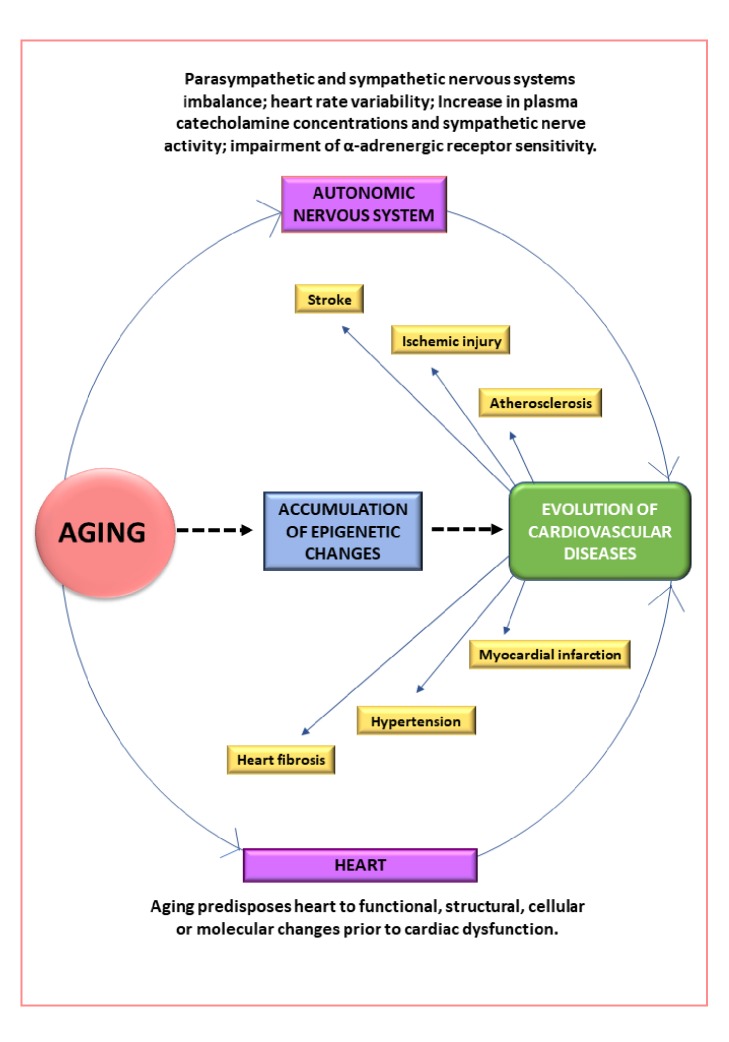
Summary of the complex alterations in aging-induced cardiovascular diseases.

In order to further the discussion of aging-induced CVDs, atherosclerosis and stroke were selected in this review. The understanding of atherosclerosis's pathogenesis requires extensive investigation of chemical mediators, cell-cell interactions, and subsequent formation of plaques. For instance, in the elderly, atherosclerotic plaques tend to be larger with increased vascular stenosis. The progressive accumulation of lipids, collagen, and calcification often occur in the plaques of the elderly as compared with younger people. Like atherosclerosis, cellular and vascular alterations are also critical to the evolution of stroke. Aging-induced endothelial dysfunction and impaired EDD have been linked to the etiology of stroke in elderly patients. Aging-induced modifications of brain microvasculature and white matter often facilitate ischemic brain damage. The alterations in neuronal conductivity by axolemma and white matter dysfunction could increase vulnerability to stroke. As shown in Figure 2, this review summarizes complex alterations that link aging to CVDs.

In perspective, the course of normal aging could be altered by physiological conditioning or pharmacological intervention. Some of the aging biomarkers, cellular and molecular targets that were identified in this review, could facilitate the diagnosis of CVDs in patients and stimulate the development of new drugs. It is possible that a given CVD and treatment pair could interfere with aging processes and promote longevity. Considering genetic and epigenetic contribution to aging processes, there may be a necessity for an individualized medicine to overcome varying responses among patients. In summary, interdisciplinary research in different aspects of aging and CVDs is crucial to new drug discovery and the promotion of knowledge-based treatment in the future.
